# Path (un)predictability of two interacting cracks in polycarbonate sheets using Digital Image Correlation

**DOI:** 10.1038/srep32278

**Published:** 2016-08-31

**Authors:** J. Koivisto, M.-J. Dalbe, M. J. Alava, S. Santucci

**Affiliations:** 1 Aalto University, Department of Applied Physics, PO Box 14100, 00076 Aalto, Finland; 2 Laboratoire de Physique de l’Ecole Normale Supérieure de Lyon, UMR CNRS 5672, Université de Lyon, 69634 Lyon Cedex 07, France; 3 Institut Lumière Matière, UMR5306 Université de Lyon 1-CNRS, Université de Lyon, 69622 Villeurbanne, France; 4 Department of Civil and Environmental Engineering, Massachusetts Institute of Technology, Cambridge, Massachusetts, USA

## Abstract

Crack propagation is tracked here with Digital Image Correlation analysis in the test case of two cracks propagating in opposite directions in polycarbonate, a material with high ductility and a large Fracture Process Zone (FPZ). Depending on the initial distances between the two crack tips, one may observe different complex crack paths with in particular a regime where the two cracks repel each other prior to being attracted. We show by strain field analysis how this can be understood according to the principle of local symmetry: the propagation is to the direction where the local shear - mode *K*_*II*_ in fracture mechanics language - is zero. Thus the interactions exhibited by the cracks arise from symmetry, from the initial geometry, and from the material properties which induce the FPZ. This complexity makes any long-range prediction of the path(s) impossible.

Fracture is a quintessential cross-disciplinary problem, in that what happens in materials on the atomistic scale becomes important at the macroscopic scale. The understanding of crack dynamics and stability has made enormous progress due to practical approaches to computational structural mechanics and materials design for stability. On the fundamental side, advances have been made e.g. in atomistic first principles simulations, and basic experiment using various techniques to follow slow and fast fracture, and also to analyze a posteriori the question “what happened”.

The coarse-grained approach of fracture mechanics seeks an answer by solving the elasticity problem for a specimen under loading, and then postulating a criterion for the stability of a defect - a crack or a notch in a prepared experiment. An example of this is the Griffith’s argument that connects the crack advancement to the fracture toughness of a material by comparing the elastic energy released by crack propagation to the energy dissipated by new crack surface formation. This illustrates in a simple fashion how the macroscopic material properties and predictability connect physics at lower scales. The original idea has been extended to ductile (Dugdale-Irwin-Barenblatt argument) and quasi-brittle materials, both of which introduce a length-scale, the size of the so-called Fracture Process Zone (FPZ) that arises by coarse-grained processes from a smaller scale.

Real materials are rarely extremely brittle, which means that cracks grow and interact, before the change to unstable dynamics takes place. The paradigm for the propagation has been for a long while that of “local symmetry”. This means according to Linear Elastic Fracture Mechanics (LEFM) selecting the direction where the stress intensity factor *K*_*II*_ = 0, or the shear stresses vanish^1^. This equals the criterion of the maximum tangential strain, MTSN, known from the 70’s^2^. The MTSN criterion is based on the St. Venant’s maximum normal strain theory. The thought-experiment that one usually does in guessing in a two-dimensional case about where a crack will go equals at looking at the dynamics of a point, the crack tip. Thus one considers the next direction to which the tip will propagate to according to such symmetry principles. In real materials, where LEFM does not hold in general the challenge is to formulate the dynamical law for slowly growing cracks: where does a local symmetry hold, such that it can be used for crack path prediction, in the presence of the FPZ?

Recently the applicability of LEFM in both quasi-static and dynamical crack propagation has been actively studied^3^,^4^,^5^,^6^,^7^,^8^. A major reason is given by advances in image analysis methods e.g. ref. 9. We consider here a paradigm of complexity for interacting cracks: a thin-sheet geometry with two initial notches, Fig. 1, studied recently^10^. In the LEFM-limit this already presents an interesting case, where the two crack tips exhibit an attraction^5^. We test samples of polycarbonate sheets. The material has a convenient feature in that one can make a clear distinction of elastic and near-tip plastic regimes^11^. In the spirit of Dugdale-Irwin theory, we follow the advance of the tips, when we are monitoring the tip of the plastic zone. The experiment (mode I, constant loading velocity, see Materials and Methods) shows consistently that for curved cracks the cracks and the plastic process zones propagate along the direction of maximum tangential strain. In general, there are three regimes that can be identified: the initial one with no interaction (Δ *y* = 0), repulsion (Δ *y* > 0) and, finally attraction (Δ *y* < 0). Interestingly the repulsion starts much before the attraction, in contrast to what is seen in the LEFM or purely elastic material case^5^. That the two cracks exhibit both initial repulsion and a later attraction is logically consistent with the *K*_*II*_ = 0 argument again^12^,^13^. A snapshot for a typical experiment is depicted in Fig. 2, at a point in which both the FPZ zones have grown and the cracks are interacting with each other. Figure 2 shows an example of two large plastic zones attracting each other just before sample starts to buckle out of plane. The general situation is shown in Fig. 3, where the trajectories of both cracks are shown in an example.

## From Repulsion to Attraction

The turning point (from “away from each other” to “towards each other”) of the cracks gives one measure of the regimes of crack repulsion and attraction. In this paper we test some other measures for the turning point. Figure 4 shows the characteristic times of selected features as a function of initial notch separation distance. The turning time *t*_*turn*_ depicted as blue dots is defined as when repulsion turns to attraction


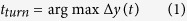


where Δ*y* is the distance between upper and lower FPZ tips. *t*_*turn*_ is thus the maximum FPZ tip vertical distance during the experiment. The time of turning correlates with the strain rate maximum in y-direction *ε*_*yy*_ (Materials and Methods) shown as red triangles and with when maximum tangential strain changes direction (maximum at 0 degree angle) depicted as green circles. For short initial separations the strain measurement indicated turning points either slightly precede or are the same as the optical measurement. For large initial distances, the plastic zones develop naturally and the strain measurement indicate a turning point which precedes the optical one actual turn. Figure 4 thus illustrates the effect of under developed PZ for small *L* and fully developed PZ for large *L*. Differences in the Fig. 4 between the grayscale and strain measurements can be explained by FPZ “inertia”. The FPZ does not follow (in how it develops as the crack advances) the natural crack growth path due to the work needed to create the new FPZ in a material with finite viscosity.

The FPZ are repelling each other when measured from grayscale images while the strain is already showing attraction. The FPZ does not turn but does it later. Note that usually in the LEFM -based theories the *stress* creates the FPZ; We are measuring *strain*, not directly the stress. In summary, the quantities show some difference which can be explained by how the turning time is defined. The “turning” is a slow process: the FPZ zone of the crack propagating to one direction has to advance a while to the new direction before the maximum y-distance can be observed, see Equation 1, when comparing this that of strain rate, which is an instant measure. In other words, the crack and the FPZ have inertia. The most interesting result in Fig. 4 is the time when MTSN changes direction. This is the time when local symmetry predicts that the crack will change direction. This also coincides with the time when the strain *yy*-component is at maximum. By definition, 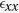 and 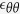 are equal when *θ* = 0.

## Local Symmetry Argument

**Figure 1:**
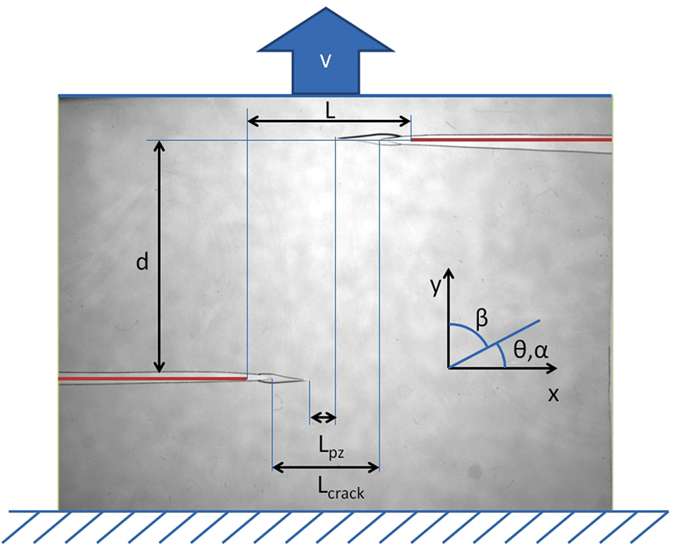
The snapshot shows the sample geometry. Initial notches are depicted with red and their initial distances are labeled as L for horizontal and d for vertical. The tension is applied with a constant velocity of v = 0.02 mm/s. *β* is the angle between the applied tension and initial notches. *α* is the complement of *β. θ* refers to the angle of the propagating plastic/yielding zone respect to horizontal axis. For completeness, the distances between the cracktips (*L*_*crack*_) and FPZ tips (*L*_*pz*_) are also shown.

Next we show in detail that the crack growth direction obeys the local symmetry argument. This argument is usually taken as granted as its roots are in the 1960’s. To be more precise there are a few rigorous treatments to this fundamental property. For a review of growth laws see e.g. refs 14 and 15. In our geometry *α* is the angle between horizontal axis and crack propagation direction, initially *α* = 0°, see Fig. 1 for details. Cottrell and Rice^1^ conclude in their paper that maximum tangential stress (or similar) criteria essentially means that the stress intensity factor *K*_*II*_ = 0 at the propagation direction. They note that this holds for small propagation angles *α* < 15°. This is called the local symmetry argument. It can be also shown that in the case of stationary cracks, the *K*_*II*_ component changes sign when the ratio between horizontal and vertical crack distances is changed^12^. In other words, the shear component sign change is related to the observation of first repulsion and then attraction as the crack propagates. Note that in ref. 12 the crack is static and the *K*_*II*_ is measured as when the crack would grow horizontally with *α* = 0°. We test the more general case as the cracks grow and interact.

Comparison between crack propagation criteria is given by e.g. Maiti *et al*.^2^, where four methods are considered: i) maximum tangential stress, ii) maximum tangential strain, iii) maximum tangential principal stress and iv) strain energy density criterion. From their discussion one can conclude that when an angle of a slit crack *α* ≤ 15°, all methods give the same result for crack propagation angle within 1 degree. The local symmetry argument essentially claims that the FPZ or yield zone will propagate to the direction where Mode II component vanishes or is minimized^1^. This can be measured by finding the location of maximum tangential strain. As the stress of a homogeneous material is difficult to measure, the approximation is made that the maximum tangential strain criterion estimates the same propagation angle as the maximum tangential stress.

Next we analyze the strain fields for a few cases with different methods showing different propagation angles. First the strain is measured at the circle around the FPZ tip (see Materials and Methods). Second the strain field is depicted at various time steps showing attraction and repulsion for two different geometries. Finally we compare the propagation angles obtained by optical observation (see also ref. 10) and strain measurements. Figure 5 depicts the cross component of the strain tensor *ε*_*xy*_ similarly to ref. 16, that is the LEFM expression for the component is used to fit the data. The strain is measured on a circle at a 1 mm radius from the FPZ tip. The theoretical line is plotted as if the crack would propagate in 30 degree angle in a repulsion case, that is using a 30 degree offset (rotation). This shift is an example of a very strong repulsion. In the experiments, much less strong repulsion (smaller angles) is observed.

**Figure 4:**
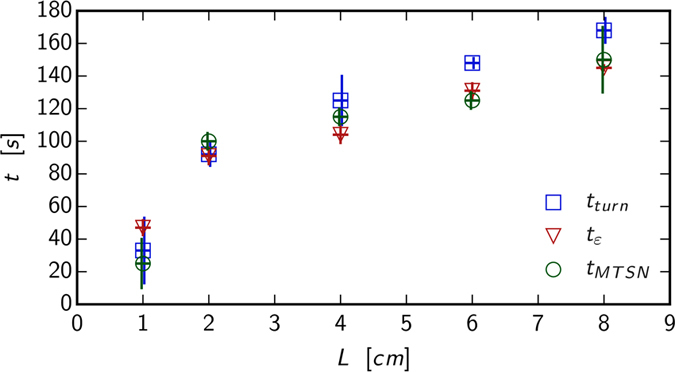
(**a**) Characteristic times for experiments with *d* = 40 mm. *t*_*turn*_ correspond to the turning time extracted from the tip trajectory, as the time when it goes from repulsion to attraction. *t*_*ε*_ corresponds to the time when the strain rate 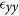 is maximum. *t*_*MSTN*_ corresponds to the time when the maximum tangential strain changes direction. Errorbars are the measurement uncertainties projected into time axis. Vertical errorbars are shifted by linewidth for clarity.

Figure 6a–c depict the tangential strain just at the beginning, repulsion and attraction for experiment with *d* = 4 cm initial separation. The FPZ tip as the origin is marked with a black cross. Figure 7 shows the same quantity with *d* = 1 cm initial separation. Figure 8 shows the comparison between maximum tangential strain MTSN and FPZ propagation angle defined from grayscale images. The dotted line shows the path of the maximum tangential strain where the elongation is the greatest along the arc of the circle with FPZ tip as a center. The solid line is the crack propagation angle *θ* = 0.5 Δ*y*/Δ*x* where Δ*x* and Δ*y* are the horizontal and vertical distances between the two FPZ tips defined from the grayscale images^10^, computed such that the asymmetry in the tensile test (fixed bottom clamp) is removed. The propagation angle seems always to be a bit less than the maximum strain direction. This is due again to the non-elastic properties of the material as was noted in the context of Fig. 4. This is also consistent with LEFM predictions and propagation angle analysis. When the crack tries to turn more than 15°, more higher order terms are needed and a simple maximum tangential strain estimate fails. That is, the theoretical estimates mentioned above also show that the propagation angles with simple maximum arguments are only valid for small propagation angles.

**Figure 5:**
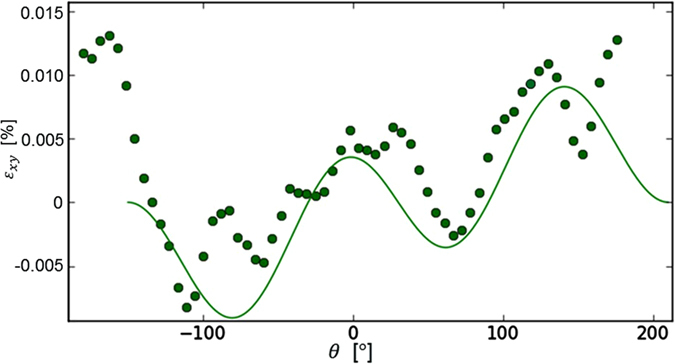
Example of shear strain from a measurement (dots) is close to the theoretical value (line). The theoretical value is from LEFM with 30 degree shift (offset to the angular values) indicating that crack would turn to 30 angle. The data is from experiment with *L* = 4 cm horizontal and *d* = 2 cm vertical separation when the FPZ interaction is very strong.

**Figure 6:**
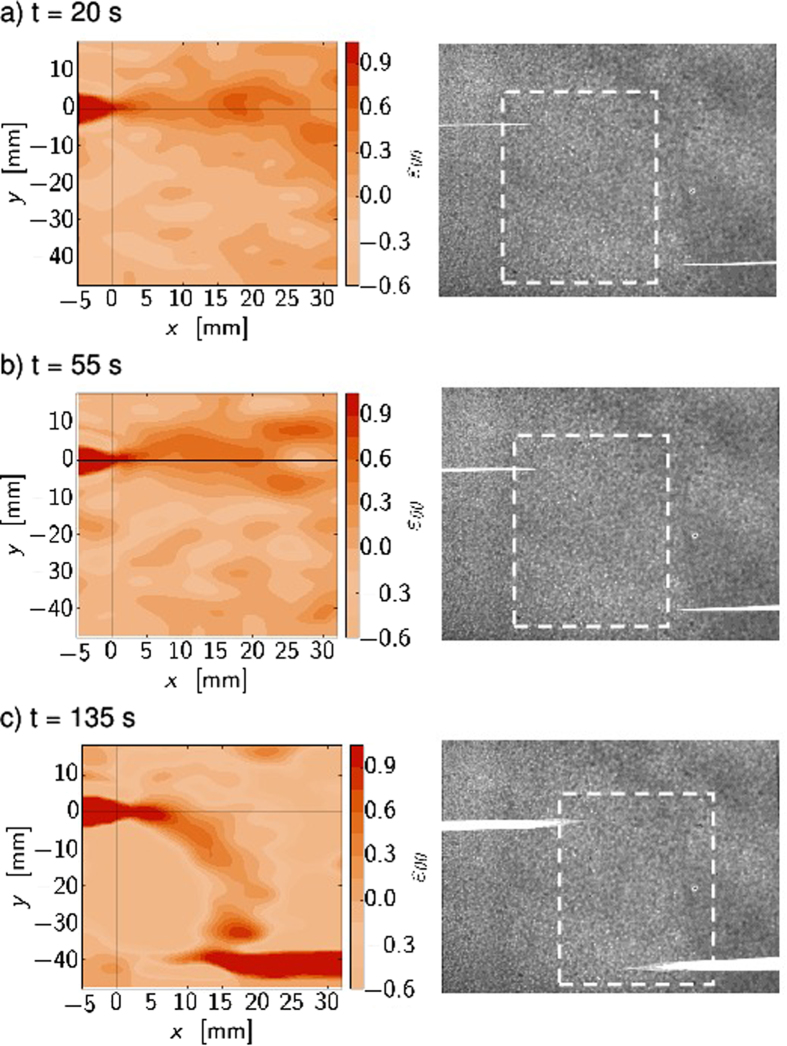
A comparison between tangential strain ( 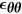) map and gray scale crack locations shows how the strain developed ahead of the crack tip for three different time steps. The geometry is with *d* = 4 cm vertical and *L* = 4 cm horizontal initial separation of the cracks. The solid black lines show the origin where the FPZ tip is located. The dashed box shows the location where the strain field is obtained. In (**a**) at *t* = 20 s the strain is still small and looks symmetric. In (**b**) *t* = 55 s *K*_*II*_ component has positive values based on the geometry^17^,^18^ which means that the crack turns counterclockwise. This is seen as repulsion: the strain maximum is above the horizontal line. At the end of the experiment *t* = 135 s in (**c**) the analytical solution gives negative values: crack turns clockwise and strain maximum is below the horizontal line.

**Figure 7:**
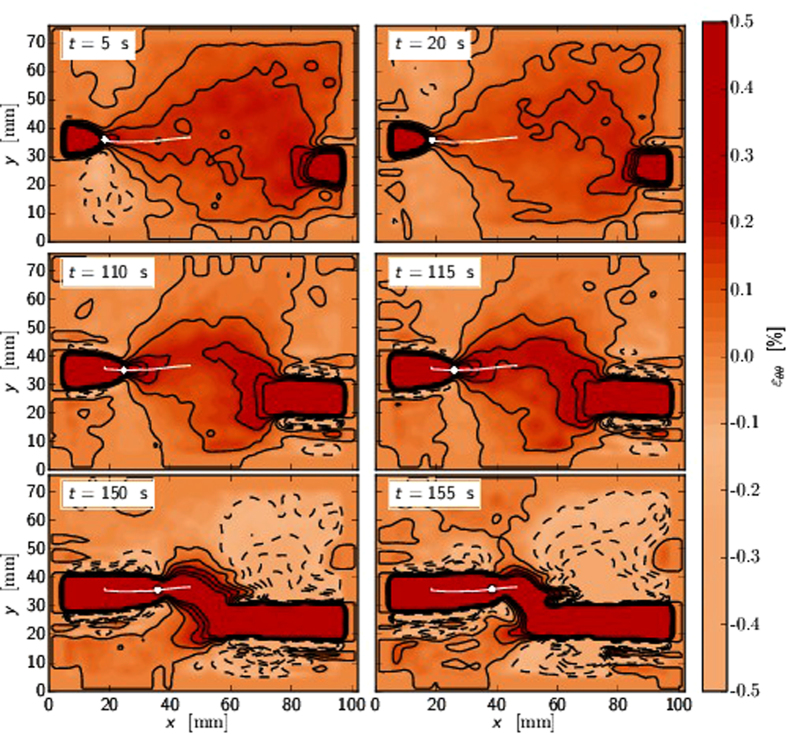
The figure represents the tangential strain similarly to Fig. 6 but with larger inital horizontal separation *L* = 60 mm and smaller initial vertical separation *d* = 10 mm. The white dot is at the FPZ tip propagating left to right along the path depicted by the white line. The other FPZ is seen as a dark red blob at *x* = 80 mm, *y* = 2 mm, *t* = 5 s propagating right to left. Top row, *t* = 5 s and *t* = 20 s: At the start of the experiment the tangential strain fields look symmetric and the crack propagates straight. Middle row, *t* = 110 s and *t* = 115 s: The strain fields look asymmetric. The maximum tangential strain directions point away from each other. The strain fields start to interact strongly and the repulsion is strong (c.f. the weaker repulsion in *d* = 4 cm case). Bottom row, *t* = 150 s and *t* = 155 s: The FPZs are close to each other and start to attract each other. This is seen best at the supplemental video online.

**Figure 8:**
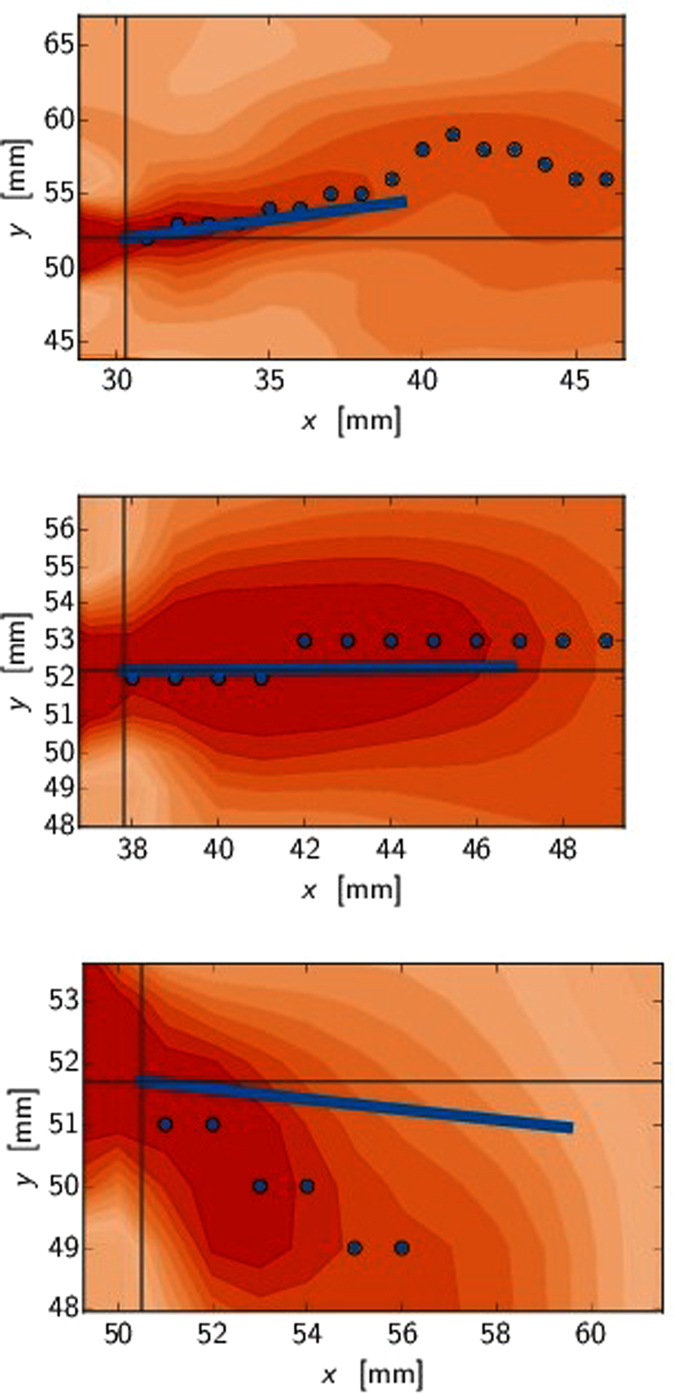
Tangential strain for 3 cases show the change in strain fields near the FPZ tip marked with black cross. The data is the same as in Fig. 6 (*d* = 4 cm, *L* = 4 cm). The other crack is propagating from right to left below the imaging area. The dotted line shows the maximum in tangential strain. The solid line is the FPZ propagation direction with slope 0.5Δ*y*/Δ*x* defined similarly as in ref. 10. Top: (*t* = 55 s) In the repulsive regime the strain maximum is slightly above the *y* level of the FPZ tip near the tip. Center: (*t* = 100 s) Near the turning point the slope and the maximum are almost horizontal. Figure 4 depicts the time instance (here *t* ≈ 100 s) when the slope changes from positive to negative for all experiments. Bottom: (*t* = 155 s) In the attractive regime the strain maximum is clearly pointing downwards.

To recapitulate, cracks tend to propagate along paths where shear strain vanishes: *ε*_*xy*_(*θ* = 0) = 0 and at the simplest one may note that in Fig. 5 the *ε*_*xy*_(*θ* = 0) is positive. This means that the crack is not going to propagate straight but turns towards positive angles (counter clockwise). This is a simple version of a complicated process that is described in detail in ref. 17 where multiple criteria for crack propagation and strain field values are compared in en passant geometry.

## Summary

Here, we have shown that the crack propagation direction can be obtained by using optical displacement measurement in thin sheets by considering the strain fields at an appropriate distance ahead of the crack tip and the FPZ. In polycarbonate sheets, FPZ tip zone can be treated as the crack tip and thus the MTSN and local symmetry criteria used. The slightly curved cracks obey the local symmetry argument. The cracks interact, such that the shear deformations affect the respective FPZ, their development, and induce an early-stage - “prior to meeting” - repulsion contrary to the purely elastic case. In materials with heterogeneities and with yielding, we expect that with the same key argument, measuring the Mode II (shear) component, one can provide local estimates of the near-future crack propagation path. This result is of importance in heterogeneous materials due to the memory implied: one can construct a probability map of possible crack propagation directions ahead of the crack/FPZ tip. However, due to the mutual interaction(s) of the two cracks here, the predictability does not extend to beyond a small scale that changes with propagation as it depends on the exact crack and FPZ geometry. An example would be the prediction of the precise point at which the initial repulsion turns into attraction due to the mutual propagation of the cracks and the evolution of the respective FPZ. It would be interesting to repeat these experiments for other materials than polycarbonate, with its large FPZ. One question would indeed be how big a process zone is needed to observe noticeable repulsion.

## Materials and Methods

The material used in this study is a polycarbonate sheet of 100 × 120 × 0.13 mm^3^. The sample is notched from two sides symmetrically with two tunable parameters: horizontal *L* and vertical *d* notch distances (Fig. 1). The vertical distance is varied from *d* = 10 mm to 40 mm and the horizontal distance is varied *L* = 10, 20, 40, 60, 80 mm. The experiment set is repeated two times, with and without tracers. In the latter case, the transparent sample is painted with inert water based paint. This is to help either the edge detection (no tracers) or digital image correlation algorithm (with tracers). A snapshot (Fig. 1) shows the sample geometry. Initial notches are depicted with red. *β* is the angle between the applied tension and initial notches. *α* is the complement of beta. *θ* refers to the angle of the propagating plastic zone respect to horizontal axis.

The sample is attached to a standard tensile testing machine, Instron Electropuls 1000. Figure 1 depicts the orientation of the sample is such that the primary stress and strain are perpendicular to the notches. The effective testing plane is 100 × 100 mm^2^ due to 10 mm material reserved for clamping on top and bottom. The testing machine imposes a constant displacement speed of *v* = 0.02 mm/s for the upper crosshead. The machine stores the force-displacement data with a frequency of *f* = 500 Hz. The images are recorded by a standard digital grayscale camera Dalsa HM1024. The analysis of the experimental data relays on three methods. First the standard force-displacement data is obtained from the tensile testing machine. Second, the plastic zone tip location is obtained from conventional image analysis (see ref. 10 and references therein). Finally the displacement field is obtained using digital image correlation algorithm (DIC)^19^,^20^,^21^,^22^. The algorithm and its accuracy is explained in detail in ref. 23 and is used also in ref. 24.

The DIC algorithm compares two gray scale images together and calculates a displacement map between the two images. The algorithm at hand uses 2 dimensional 3rd degree b-splines for the mapping with one node in a 32 × 32 pixel (0.3 × 0.3 mm) region interest. In a smooth linear deformation near the crack tip a 0.1 pixel (1 *μ*m) accuracy is a good starting point. In the case of strain, a 0.1 pixel displacement in a 32 pixel distance corresponds to a strain accuracy of 0.3% which is used here. Increasing the derivation lag increases the accuracy but decreases the resolution.

The measured peak strain at the FPZ tip is around 0.9% in a 1 mm region in a 2-second time window. With this rate the material has reached its yield strain in 5 seconds. This coincides with the 5% yield strain of polycarbonate and average FPZ propagation speed of 0.2 mm/s. In a 100-second experiment the FPZ has propagated 20 mm corresponding roughly to the observed behavior.

The tangential strain (or hoop strain) 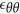 ref. 25 is a numerical derivative of displacement along the circular arc with FPZ as center. Cartesian displacements are obtained from the mentioned digital image correlation algorithm. In this paper strain 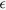 is measured in a time window of *t*_*strain*_ = 2 s. We use the word strain instead of strain rate because of simplicity.

## Additional Information

**How to cite this article**: Koivisto, J. *et al*. Path (un)predictability of two interacting cracks in polycarbonate sheets using Digital Image Correlation. *Sci. Rep.* **6**, 32278; doi: 10.1038/srep32278 (2016).

## Supplementary Material

Supplementary Movie

Supplementary Information

## Figures and Tables

**Figure 2:**
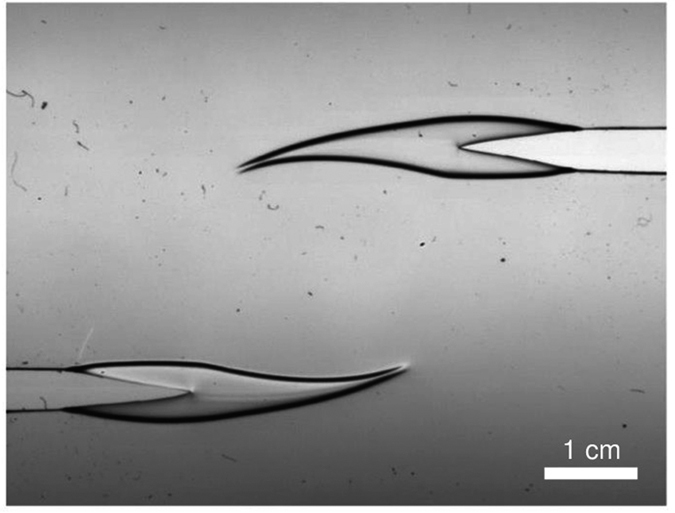
Example of two cracks with 4 × 2 cm initial separations (horizontal, vertical), which create large and visible plastic process zones that interact strongly.

**Figure 3:**
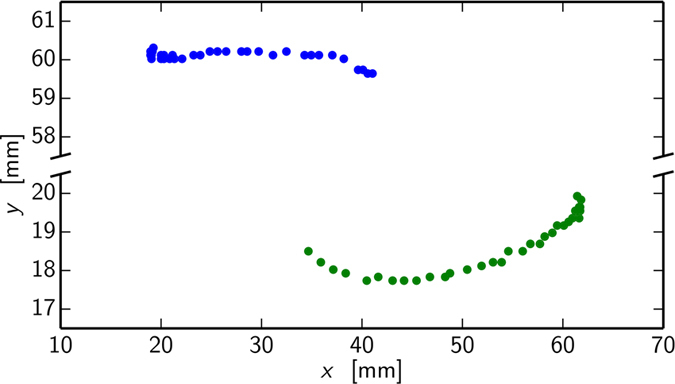
Paths of two cracks with 40 mm initial separation (L = 4 cm) have different curvatures. The coordinates are as they are seen in the camera. Notice that the scale is the same for both cracks but there is a cut in the y-axis.
