# Supplementary material for: Path (un)predictability of two interacting cracks in polycarbonate sheets using Digital Image Correlation
Source: Sci Rep. 2016 Aug 31;6:32278. doi: 10.1038/srep32278 (PMC5006083; doi:10.1038/srep32278)
Supplement: Supplementary Information [file srep32278-s2.pdf]

Supplementary Information: Path (un)predictability of two interacting cracks in polycarbonate sheets using Digital Image Correlation

J. Koivisto, M.-J. Dalbe, M.J. Alava, and S. Santucci

Legend for Supplementary Movie 1:

Supplementary Movie 1 (d1cm.avi): The video shows an example of crack growth for $d = 1$ cm.
